# Targeting autophagy enhances the anti-tumoral action of crizotinib in ALK-positive anaplastic large cell lymphoma

**DOI:** 10.18632/oncotarget.4999

**Published:** 2015-08-17

**Authors:** Géraldine Mitou, Julie Frentzel, Aurore Desquesnes, Sophie Le Gonidec, Talal AlSaati, Isabelle Beau, Laurence Lamant, Fabienne Meggetto, Estelle Espinos, Patrice Codogno, Pierre Brousset, Sylvie Giuriato

**Affiliations:** ^1^ Inserm, UMR1037 CRCT, F-31000 Toulouse, France; ^2^ Université Toulouse III-Paul Sabatier, UMR1037 CRCT, F-31000 Toulouse, France; ^3^ CNRS, ERL5294 CRCT, F-31000 Toulouse, France; ^4^ Phenotyping Service, INSERM-US006 ANEXPLO/CREFRE, Toulouse, France; ^5^ INSERM/UPS - US006/CREFRE, Service d'Histopathologie, CHU Purpan, Toulouse, France; ^6^ INSERM UMRS 1185; Faculté de Médecine Paris Sud, Le Kremlin-Bicêtre, France; ^7^ Université Toulouse III - Paul Sabatier, Toulouse, France; ^8^ Department of Pathology, IUCT, Toulouse, France; ^9^ Institut Necker Enfants-Malades, INSERM U1151-CNRS UMR 8253, Paris, France; ^10^ European Research Initiative on ALK-related malignancies (ERIA)

**Keywords:** anaplastic large cell lymphoma, NPM-ALK, autophagy, crizotinib, cytoprotection

## Abstract

Anaplastic Lymphoma Kinase-positive Anaplastic Large Cell Lymphomas (ALK+ ALCL) occur predominantly in children and young adults. Their treatment, based on aggressive chemotherapy, is not optimal since ALCL patients can still expect a 30% 2-year relapse rate. Tumor relapses are very aggressive and their underlying mechanisms are unknown. Crizotinib is the most advanced ALK tyrosine kinase inhibitor and is already used in clinics to treat ALK-associated cancers. However, crizotinib escape mechanisms have emerged, thus preventing its use in frontline ALCL therapy. The process of autophagy has been proposed as the next target for elimination of the resistance to tyrosine kinase inhibitors. In this study, we investigated whether autophagy is activated in ALCL cells submitted to ALK inactivation (using crizotinib or ALK-targeting siRNA). Classical autophagy read-outs such as autophagosome visualization/quantification by electron microscopy and LC3-B marker turn-over assays were used to demonstrate autophagy induction and flux activation upon ALK inactivation. This was demonstrated to have a cytoprotective role on cell viability and clonogenic assays following combined ALK and autophagy inhibition. Altogether, our results suggest that co-treatment with crizotinib and chloroquine (two drugs already used in clinics) could be beneficial for ALK-positive ALCL patients.

## INTRODUCTION

Anaplastic Large Cell Lymphoma (ALCL) is an aggressive form of malignant lymphoma of T/null lineage, which occurs mostly in children and young adults. Two systemic forms of ALCL have been defined according to the presence or absence of aberrant anaplastic lymphoma kinase (ALK) expression [1, 2]. The identification of ALK and its role in the pathogenesis of ALCL were originally described in 1994 by Morris et al. [3]. Since then, it has become increasingly clear that ALK is a prevalent oncogene that is aberrantly expressed in a variety of tumors, including some B-cell lymphoma (DLBCL), inflammatory myofibroblastic tumors (IMT), some non-small-cell lung cancers (NSCLC), renal carcinoma (RCC), colorectal carcinoma (CRC) and neuroblastoma (NB) [4–6]. In the case of ALCL, ALK is mainly activated as a consequence of a chromosomal translocation whereby the oligomerization domain of the nucleophosmin (NPM) gene is juxtaposed to the kinase domain of ALK. The resultant NPM-ALK fusion protein is constitutively active, and has been described in different cell and mouse models for NPM-ALK tumorigenesis [4, 7–10]. In NSCLC, an inversion event fuses the echinoderm microtubule-associated protein-like 4 (EML4) gene to ALK. Other less represented chromosomal abnormalities involving the ALK gene have also been described both for ALCL and NSCLC [5]. In addition, ALK activation in cancer can also arise through overexpression and mutation of full-length ALK [6].

ALK-expressing tumors are sensitive to treatment with small molecule inhibitors [11–14]. Among these, crizotinib is a potent ATP-competitive inhibitor of ALK and c-Met [15]. It is already used in the clinic for the treatment of late stage and metastatic cases of ALK-positive NSCLC, and promising results have accumulated concerning its use in the treatment of IMT [16, 17] and recurring/refractory ALCL [17, 18]. However, as has been reported for other tyrosine kinase inhibitors, escape mechanisms to overcome the effects of crizotinib have been described in ALK-positive NSCLC and ALCL patients [6, 13, 18–23]. These mainly occur through ALK tyrosine kinase domain punctual mutations, ALK gene amplification and/or activation of compensatory signaling pathways and more than one mechanism can develop simultaneously within the same tumor [13, 24]. Thus, to circumvent resistance, second generation ALK inhibitors (AP26113, LDK 378, ASP3026, CH5424802) have been developed [5], and new combined therapies have also been proposed using a non-ALK-targeting drug alongside an ALK inhibitor [25, 26]. Despite this, our understanding of crizotinib resistance mechanisms at both the cellular and molecular levels needs improvement in order to develop better treatment options. For instance, tumor cell autophagy has been proposed as a new target for overcoming resistance to tyrosine kinase inhibitors [27–30], yet its role in crizotinib-treated ALCL has never been studied.

Autophagy is a highly-conserved catabolic pathway used by the cell to degrade and recycle its own constituents [31]. The autophagy process involves first the formation of an isolation membrane, which elongates and closes in on itself to isolate unwanted cytoplasmic components such as damaged or obsolete organelles and toxic protein aggregates within a double-membraned structure called an autophagosome. Autophagosomes then dock and fuse with lysosomes, where acidic hydrolases degrade the “cargo”, therefore assuring a constant cytoplasmic quality control. All of the steps of this process are tightly regulated [32–34].

Malfunctioning autophagy is observed in many human diseases (including neurodegenerative diseases, infectious diseases, heart diseases and diabetes) [35]. In cancer development, autophagy plays a dual role [36–39]. During the initial stages of tumor development it exerts anti-tumoral effects, essentially by removing damaged mitochondria, preventing ROS accumulation, tissue damage, inflammation and genomic instability [40]. Conversely, once the tumor is formed it fuels tumorigenesis by delivering energy in a metabolically stressed environment [41].

Emerging evidence shows that these dual functions of autophagy, in promoting either death or survival mechanisms, are also observed in therapeutically-challenged tumor cells. Indeed, some tumor treatments have been associated with autophagy-mediated oncoprotein degradation [42, 43]. Other compounds have been shown to induce autophagy-mediated cell death [44–46] or autophagy-mediated immunogenic cell death [47]. Conversely, survival autophagy mechanisms have also been observed in different studies upon either chemo-, radio- or targeted therapies [27, 29, 48–50] and inhibiting autophagy in these contexts has been proven to enhance treatment efficiency [51, 52]. Thus, whether to use autophagy inducers or inhibitors to optimize a given cancer treatment is clearly a matter of context [50].

In this study we sought to investigate whether autophagy activation acts as a tumor survival mechanism to overcome ALK oncogene inactivation in ALCL cell lines and whether disabling autophagy may represent a clinical benefit for ALCL patients.

## RESULTS

### ALK inactivation induces autophagy in ALK-positive ALCL cell lines

**Figure 1:**
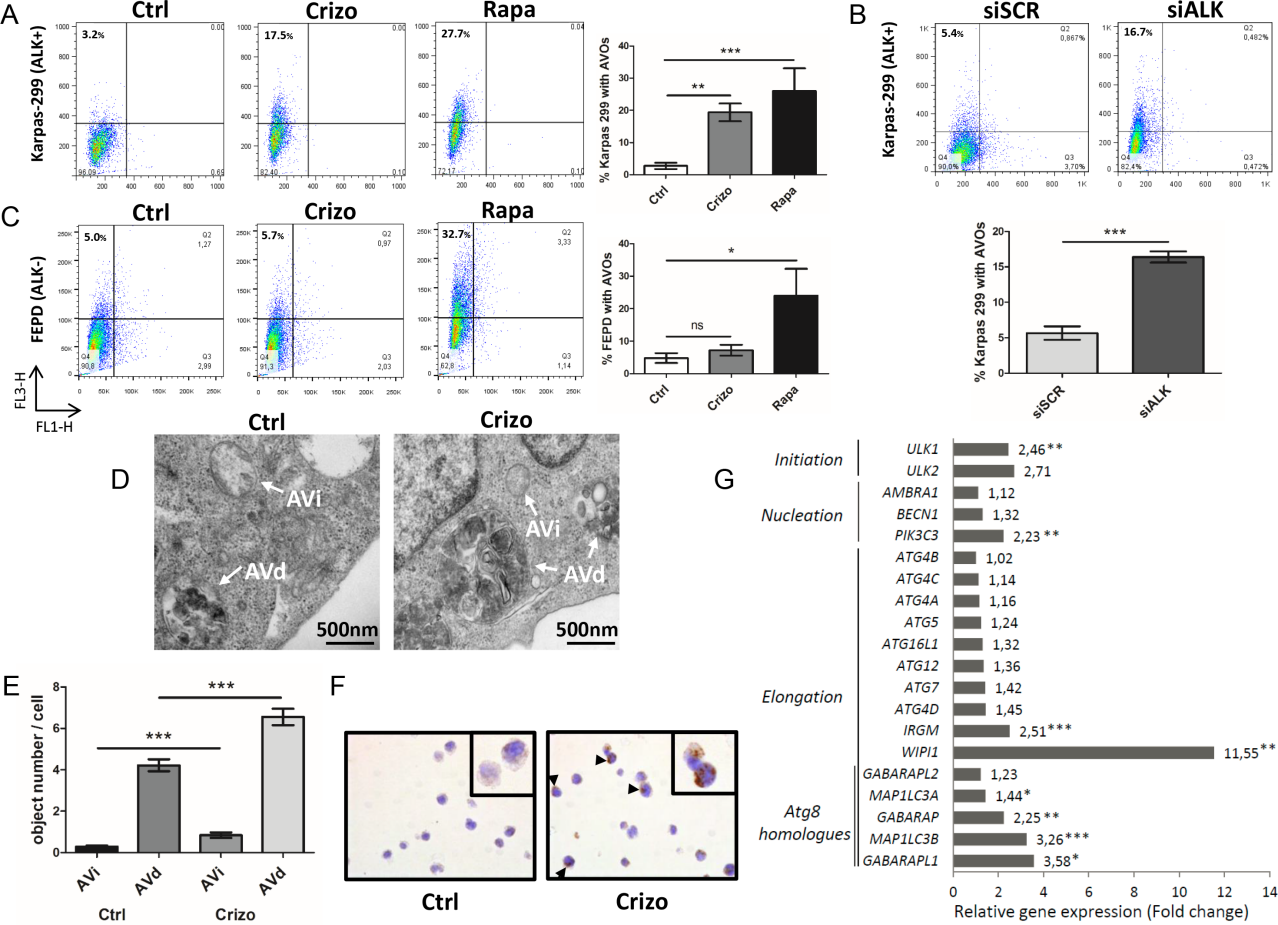
Induction of autophagy upon ALK inactivation in NPM-ALK-positive Karpas-299 ALCL cells **A.** Acridine orange flow cytometry staining was performed to detect the formation of acidic vesicular organelles (AVOs) following crizotinib (Crizo) (500 nM, 24 h) or rapamycin (Rapa) (100 nM, 24 h) treatment, compared to control cells (Ctrl). FL1-H indicates green color intensity (cytoplasm and nucleus), while FL3-H shows red color intensity (AVOs). The percentage of AVOs is displayed in the left upper quadrants. Representative flow diagrams are shown. Data on graph represent mean AVOs quantification ± SD from three independent experiments. Statistical analysis was performed by unpaired *t*-tests; ***p ≤ 0.001; **p ≤ 0.01. **B.** AVOs development and quantification were determined, as indicated in (A), following transfection for 72 h with ALK-targeted siRNA (siALK) or scramble siRNA (siSCR). **C.** AVOs quantification was determined, as indicated in (A), for untreated, crizotinib-treated (500 nM, 24 h) and rapamycin-treated (100 nM, 24 h) ALK-negative FEPD ALCL cells. Mean AVOs percentages are represented ± SD, quantified from three independent experiments. Statistical analysis was performed by one-way ANOVA followed by the Newman–Keuls multiple comparison test; ***p ≤ 0.001. **D.** Quantification of autophagic vacuoles was performed on around 100 cells from TEM sections prepared from untreated (Ctrl) and crizotinib-treated (Crizo) (500 nM, 24 h) conditions. Characteristic double membrane autophagosomes were counted as initial autophagic vacuoles (AVi) whereas autophagosomes that had fused with vesicles originated from the endo/lysosomal compartment were counted as degradative autophagic vacuoles (AVd). Representative images at x 10,000 magnification are shown. **E.** Data represent mean vesicle number per cell ± SEM. Statistical analysis was performed by an unpaired *t*-test; ***p ≤ 0.001. **F.** LC3 immunohistochemical staining in control (Ctrl) and crizotinib-treated Karpas-299 cells (500 nM, 24 h) (Crizo). Sections were stained with anti-LC3 antibodies, and nuclei were counterstained with hematoxylin. Black arrows denote punctuate LC3 staining. Original images were produced with a leica DM4000B microscope (total magnification: x 400). **G.** Autophagy-related gene expression profile following crizotinib treatment. This selected data set was obtained using SABiosciences autophagy PCR arrays (*n* = 3). Results are expressed as fold change compared to levels measured in untreated Karpas-299 cells (set to 1). Statistical analysis was performed using unpaired *t*-tests; *p ≤ 0.05; **p ≤ 0.01; ***p ≤ 0.001.

To determine whether ALK inactivation induces autophagy, we used flow cytometry to assay the development of acidic vesicular organelles (AVOs), which are indicative of autophagy, using the lysosomo-tropic agent, acridine orange [53]. This compound accumulates in acidic compartments to form aggregates that fluoresce bright red. As a positive control, Karpas-299 cells were treated with rapamycin (100 nM, 24 h), a well-known inducer of autophagy (Figure 1A). We observed that when ALK-positive Karpas-299 (Figure 1A and 1B) or SU-DHL-1 cells (Supplemental Figure S1A and S1B) were submitted to pharmacological or molecular ALK inactivation, through crizotinib treatment or ALK-targeted siRNA transfection respectively, an increase in the red fluorescence (y-axis) was observed. This demonstrates the induction of AVOs following ALK inactivation, from 3.2 to 17.5% upon crizotinib treatment and from 5.4 to 16.7% following NPM-ALK downregulation. We confirmed the loss of NPM-ALK autophosphorylation (data not shown) and the decreased viability of the ALCL cell lines (Supplemental Figure S2A and S2B) following treatment with crizotinib at either 500 nM for Karpas-299 cells (a concentration which corresponds to the plasmatic dose measured in patients being treated for ALK tumors) or 400 nM for SU-DHL-1 cells, which are more sensitive to the drug [15]. The downregulation of NPM-ALK expression following ALK-targeted siRNA was also checked, and is shown in Supplemental Figure S3A.

To assess the specificity of AVOs induction following ALK inactivation, we used the ALK-negative ALCL cell line, FEPD, treated or not with crizotinib (500 nM, 24 h) or rapamycin (100 nM, 24 h). Rapamycin treatment induced AVOs formation, whereas crizotinib treatment did not (Figure 1C). This strongly argues for a direct causal relationship between ALK inactivation and AVOs generation in ALK-positive ALCL cell lines.

This observed accumulation of AVOs prompted us to validate that autophagy was induced using other techniques. To this end, we first checked for the presence of autophagosomes by electron microscopy. As shown in Figure 1D and 1E, we observed an increased number of double-membrane autophagosomes (shown by arrows) upon crizotinib treatment in Karpas-299 cells compared to untreated cells. ALK-inhibition increased the number of autophagosomes at both their initial (AVi) and late maturation stages (AVd), as morphologically defined in the Eskelinen review [54]. We then used immunohistochemistry to demonstrate an increased percentage of cells harboring a punctate distribution of the autophagy marker microtubule-associated protein 1 light chain 3 (MAP1LC3) [55], hereafter referred to as LC3, upon crizotinib treatment compared to untreated cells (Figure 1F and Supplemental Table 1). Finally, we investigated whether crizotinib treatment in ALK-positive Karpas-299 cells could have an effect on the expression levels of genes involved in the autophagy initiation and elongation processes. The analysis of a focused autophagy RT-PCR array showed a global increase in the expression of autophagy-related genes upon crizotinib treatment, in comparison with untreated Karpas-299 cells (Figure 1G). Strikingly, the highest significant up-regulations were found for genes that orchestrate the three crucial steps for autophagosome formation: (i) ULK1: involved in initiation, 2.46 fold change, *p* < 0.01; (ii) PIK3C3: involved in nucleation, 2.23 fold change, *p* < 0.01; (iii) MAP1LC3B: involved in elongation/closure, 3.26 fold change, *p* < 0.001; and (iv) WIPI1: involved in elongation/closure, 11.55 fold change, *p* < 0.01. We validated the increased levels of these four mRNAs and some of their encoding proteins in Karpas-299 cells in which ALK inactivation had been achieved through the use of ALK-targeting siRNA (Supplemental Figure S4). Altogether, these observations demonstrate that a loss of ALK activity is able to elicit morphological and molecular signatures specific to the autophagic process.

**Figure 2:**
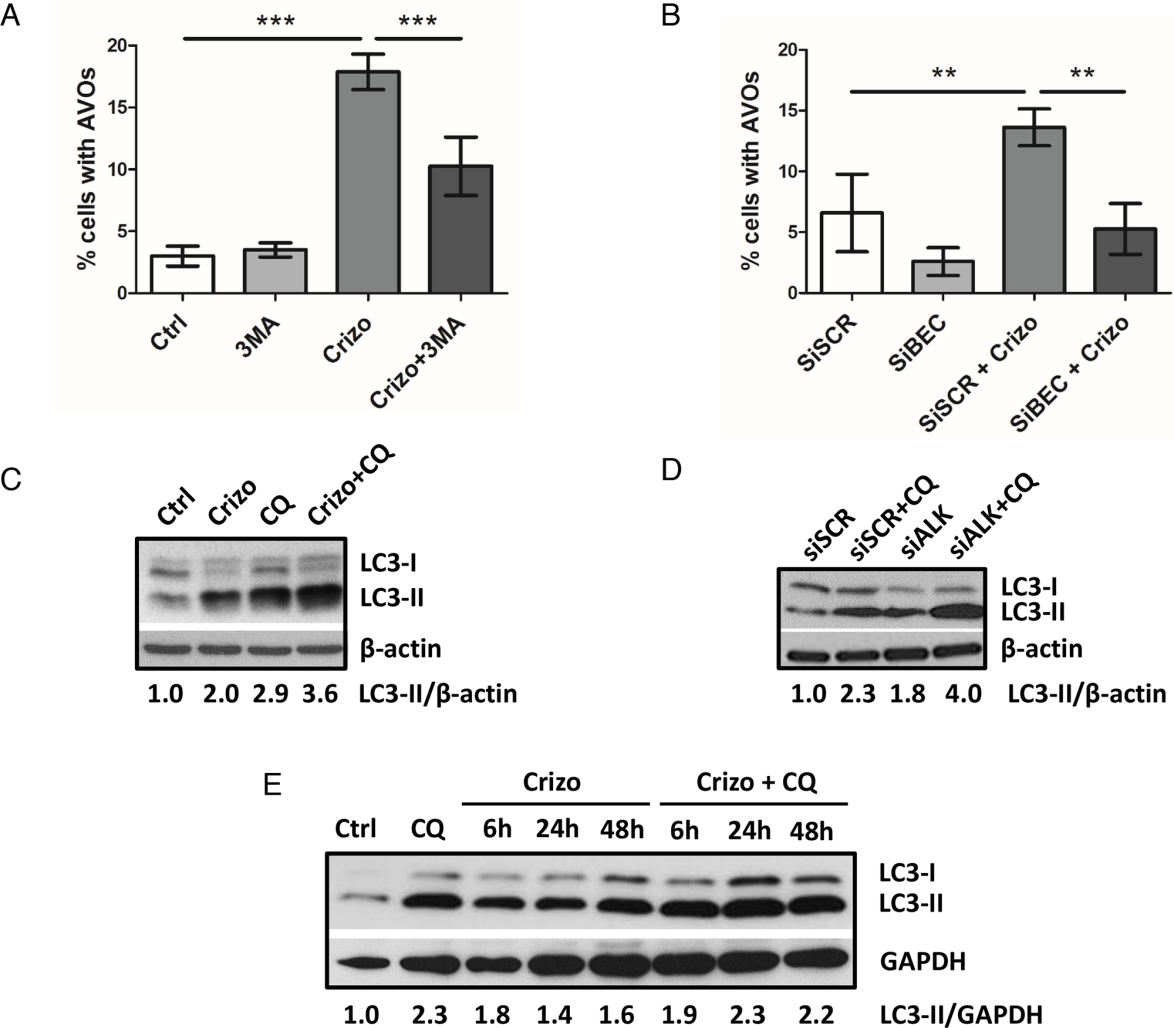
ALK inactivation increases autophagic flux in the Karpas-299 ALCL cell line **A-B.** Effect of 3-methyladenine treatment or Beclin1 knockdown on the development of AVOs in crizotinib-treated cells. Karpas-299 cells were treated or not with crizotinib (500 nM, 24 h) (Crizo). 3-methyladenine (3MA) (10 mM) was added or not 4 h before AVOs quantification by acridine orange FACS staining (A). Karpas-299 cells, transfected with either scramble (siSCR) or Beclin-1-targeted siRNAs (siBECN1), were treated or not with crizotinib (500 nM, 24 h). AVOs quantification was performed by acridine orange FACS staining (B). Data are expressed as mean values ± SD quantified from at least three independent experiments. Statistical analysis was performed by one-way ANOVA followed by a Newman–Keuls multiple comparison test; **p ≤ 0.01; ***p ≤ 0.001. **C-E.** Autophagic flux was determined in Karpas-299 cells following treatment with crizotinib (500 nM, 24 h) (Crizo) in the presence or absence of chloroquine (CQ) (30 μM, 24 h) (C). Karpas-299 cells were transfected with either scramble (siSCR) or ALK-targeted siRNAs (siALK) for 72 h and with additional treatment or not of 30 μM chloroquine (CQ) for the last 24 h (D). The kinetics of crizotinib treatment (500 nM, 6 h, 24 h and 48 h) (Crizo) was observed in Karpas-299 cells in the presence or absence of chloroquine (CQ) (30 μM, 24 h) (E). Total cell lysates were analyzed by western blotting using antibodies against LC3 and β-actin or GAPDH (as a loading control). Blots from three representative experiments are shown.

To further confirm the induction of autophagy and address the question of the activation of autophagic flux in ALK-inactivated Karpas-299 cells, we first performed acridine orange FACS analysis to monitor AVOs generation upon disruption of the autophagy process at an early stage. Vps34 and Beclin1 are two key proteins belonging to the PI3-kinase/Beclin1 complex that is required early on in the activation of autophagy. We used the pharmacological drug 3-methyladenine (3MA) to specifically inhibit Vps34 (a class III PI3-kinase), and an siRNA approach to inactivate Beclin1 (Supplemental Figure S3B) [32, 56]. As shown in Figure 2A and 2B, in both experimental settings we observed a drop in crizotinib-induced AVOs generation, from 18.9 ± 2.6% to 10.2 ± 2.3% upon 3-methyladenine addition (*p* ≤ 0.001) and from 13.6 ± 1.5% to 5.3 ± 2.1% upon Beclin1 downregulation (*p* ≤ 0.01). Taken together, these results indicate that the crizotinib-induced increase in red fluorescence is attributable to the development of AVOs associated with autophagy. We then analyzed autophagic flux using the LC3 turnover assay [57, 58]. For this, we inhibited ALK in Karpas-299 cells using crizotinib or siALK transfection, combined or not with chloroquine (CQ), a drug known to block autophagy by impairing the lysosomal degradation of the autophagic cargo. We then monitored the conversion of the cytosolic LC3 form (LC3-I, 18kDa) to the pre-autophagosomal and autophagosomal membrane-bound form of LC3 (LC3-II, 16kDa). Levels of LC3-II were higher in cells submitted to chloroquine treatment alongside ALK inactivation (for both crizotinib treatment and ALK-targeted siRNA) than in cells submitted to either treatment alone (Figure 2C and 2D). Furthermore, when we analyzed the kinetics of crizotinib treatment (after 6 h, 24 h and 48 h) in the presence or absence of chloroquine, we observed an accumulation of the LC3-II form over time (Figure 2E). Similarly, a CQ-dependent accumulation of LC3-II was observed in ALK-positive SU-DHL-1 cells treated with crizotinib (400 nM, 24 h) (Supplemental Figure S1C). Overall, these results indicate that ALK inactivation, either through pharmacological (crizotinib) or molecular approaches (ALK-targeted siRNA), induces an increase in the number of autophagosomes and autophagic flux in at least two of the most commonly used ALK-positive ALCL cell lines: Karpas-299 and SU-DHL-1.

### Synergistic loss of Karpas-299 cell viability upon pharmacological or molecular inhibition of both the ALK oncogene and the autophagic process

**Figure 3:**
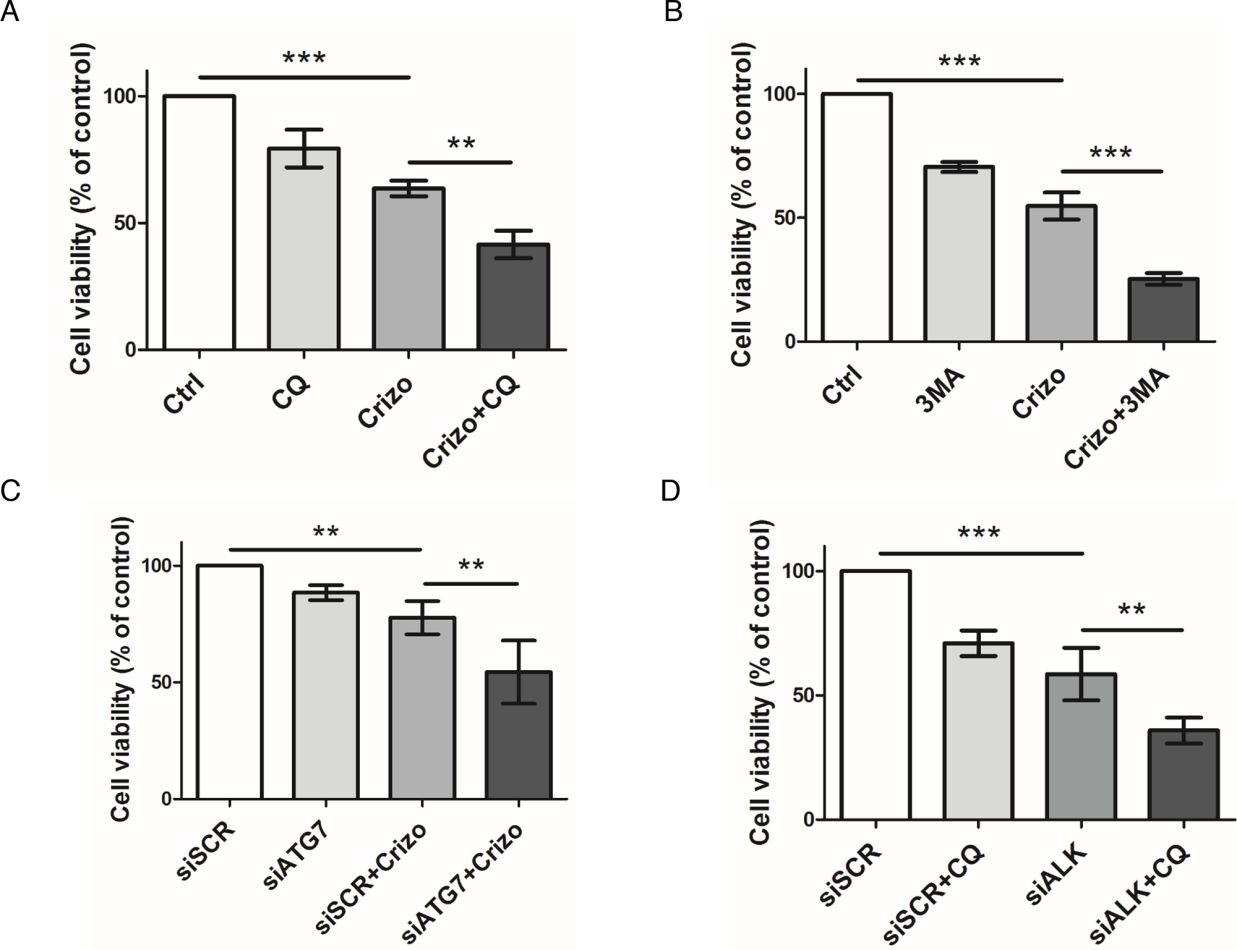
Effect of pharmacological or molecular inhibition of the ALK oncogene combined with inhibition of the autophagic process on Karpas-299 cell viability **A-B.** Karpas-299 cells were treated with crizotinib (Crizo) (500 nM) with or without chloroquine (CQ) (30 μM) (A) or 3-methyladenine (3MA) (1.25 mM) (B) for 48 h, then cell viability was determined by MTS assay. **C-D.** Karpas-299 cells were transfected with either scramble (siSCR) or ATG7-targeted (siATG7) siRNAs (C) or with scramble (siSCR) or ALK-targeted (siALK) siRNAs (D) and treated or not with crizotinib (500 nM) (Crizo) (C) or chloroquine (30 μM) (CQ) (D). Cell viability was determined by MTS assay. The graph represents mean values ± SD from three to four independent experiments. Statistical analysis was performed by one-way ANOVA followed by the Newman–Keuls multiple comparison test; **p ≤ 0.01; ***p ≤ 0.001.

**Figure 4:**
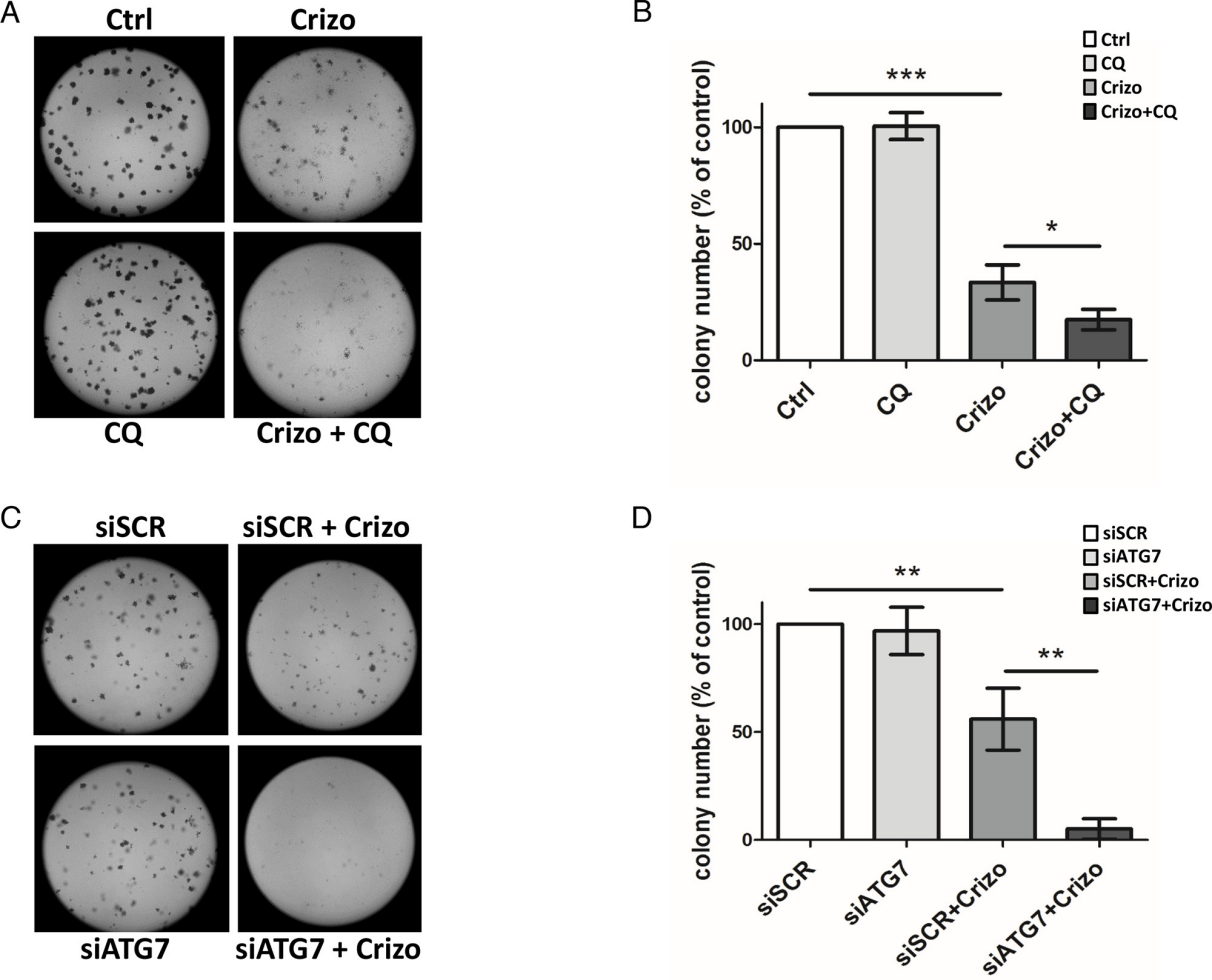
Effect of ALK oncogene inhibition combined with pharmacological or molecular inhibition of the autophagy machinery on Karpas-299 clonogenic survival **A-B.** Karpas-299 cells were treated with crizotinib (Crizo) (500 nM) with or without chloroquine (CQ) (30 μM) for 16 h. Cells were then plated onto agar plates. Colonies were detected after 6 days upon addition of the MTT reagent and were scored by Image J quantification software. Representative pictures are shown (A). Results are expressed as the number of colony forming cells per field (B). The graph represents mean values ± SEM from four independent experiments. **C-D.** Karpas-299 cells were transfected with ATG7-targeted (siATG7) or scramble (siSCR) siRNAs then treated or not with crizotinib (Crizo) (500 nM) for 16 h. Cells were then plated onto agar plates. Colonies were detected after 6 days upon addition of the MTT reagent and were scored by Image J quantification software. Representative pictures are shown (C). Results are expressed as the number of colony forming cells per field (D). The graph represents mean values ± SEM from three independent experiments. Statistical analysis was performed by one-way ANOVA followed by the Newman–Keuls multiple comparison test; *p ≤ 0.05; **p ≤ 0.01; ***p ≤ 0.001.

It is now well known that cancer cells that are able to mount an autophagic response under stress conditions are highly sensitive to chloroquine. We thus investigated, *in vitro*, how this drug would impact on ALCL cell viability following co-treatment with crizotinib. As shown in Figure 3A, we observed a decrease in ALK-positive Karpas-299 cell viability, from 63.6 ± 3.1% upon single crizotinib treatment to 41.5 ± 5.4% upon crizotinib and chloroquine co-treatment (*p* ≤ 0.01). Similar results were obtained with SU-DHL-1 cells (Supplemental Figure S1D), and when another pharmacological drug targeting autophagy was used, *i.e*. 3-methyladenine (Figure 3B). Increasing concentrations of crizotinib (0–2000 nM), combined with increasing doses of either chloroquine (0–120 μM) or 3-methyladenine (0–10 mM) (Tables 1 and 2) allowed a Chou-Talalay analysis to be performed [59], which demonstrated the synergism (defined by a combination index (CI) < 1) of co-treatment. Indeed, the CI value for crizotinib+chloroquine was between 0.85 and 0.9, indicating a slight synergism between the two drugs, and the CI value of crizotinib+3-methyladenine was between 0.3 and 0.7, showing synergism, as described by Chou (Supplemental Figure S5A and S5B) [60]. Similar results were also observed for ALK-positive SU-DHL-1 cells (Supplemental Figure S1E). Chloroquine and 3-methyladenine can also induce off-target or alternative target effects (besides autophagy inhibition) [61–63], which could have accounted for the reduced cell viability in our MTS assays. Thus, to rule out these potential effects we used siRNA targeting ATG7 to molecularly inhibit the autophagic machinery. ATG7 is a key protein involved in the maturation of the LC3 protein during the autophagosome elongation phase [32, 56]. We first checked the efficiency of ATG7 knockdown (Supplemental Figure S3C) and the effect of this on the conversion of LC3-I to LC3-II. As shown in Supplemental Figure S6, accumulation of the LC3-II form was reduced by 50% in siATG7-transfected Karpas-299 cells, both under basal and crizotinib-treated conditions, in comparison to cells transfected with scramble siRNA (siSCR). It is important to point out that under these conditions we found that ATG7 invalidation alone did not impair cell viability (Figure 3C) or cell growth on agar plates (Figure 4C). However, when combined with crizotinib treatment, it potently inhibited these two cell responses (Figures 3C and 4C). Similar results were observed with ALK-targeted siRNA combined with chloroquine (Figure 3D). Altogether, these results indicate that combined ALK and autophagy inhibition, even using diverse approaches, leads to a reduction in cell viability.

**Table 1:** Viability (%) of Karpas-299 cells after a 48 h treatment with crizotinib and chloroquine alone or in combination

	Crizotinib (nM)					
	0	125	250	500	1000	2000
**Chloroquine (μM)**						
**0**	100	93,88 ± 5,66	80,27 ± 3,31	58,75 ± 9,64	46,07± 17,67	36,03 ± 21,11
**7,5**	100 ± 5,72	82,52 ± 7,05				
**15**	96,97 ± 3,83		69,01± 13,08			
**30**	79,35± 7,36			41,53 ± 5,36		
**60**	68,56 ± 3,37				20,90 ± 2,96	
**120**	15,13 ± 5,13					

Karpas-299 cells were treated for 48 h with the indicated concentrations of crizotinib (nM) and chloroquine (μM), either alone or in combination. A constant ratio design was used for combination experiments. Viability was measured by MTS assay. Experiments were done at least in triplicate and were normalized to cells incubated without drug (100%). Data represent the mean values ± SD.

**Table 2:** Viability (%) of Karpas-299 cells after a 48 h treatment with crizotinib and 3-methyladenine (3MA) alone or in combination

Crizotinib (nM)						
	0	125	250	500	1000	2000
**3MA (mM)**						
**0**	100	83,65 ± 2,70	69,79 ± 3,38	52,48 ± 4,13	41,43 ± 3,87	37,5 ± 4,33
**0,625**	82,74 ± 3,17	62,42 ± 6,92				
**1,25**	71,23 ± 1,63		44,09 ± 3,89			
**2,5**	51,61 ± 0,38			15,81± 4,46		
**5**	31,24 ± 1,20				8,15 ± 4	
**10**	14,33± 3,82					

Karpas-299 cells were treated for 48 h with the indicated concentrations of crizotinib (nM) and 3-methyladenine (mM), either alone or in combination. A constant ratio design was used for combination experiments. Viability was measured by MTS assay. Experiments were done at least in triplicate and were normalized to cells incubated without drug (100%). Data represent the mean values ± SD.

### Reduced clonogenic potential and increased apoptosis following combined ALK and autophagy inhibition

The synergistic effect of combined ALK and autophagy inhibition on reducing ALK-positive Karpas-299 and SU-DHL-1 cell viability raised the question of whether this was due to a decrease in cell growth and/or an increase in cell death. To address this point, we first analyzed the ability of Karpas-299 cells to grow in soft agar following treatment with either crizotinib or chloroquine or both drugs in combination (Figure 4A and 4B). To confirm these results, the same experiments were also performed using siRNAs targeting ATG7 to directly impair autophagy (Figure 4C and 4D). We found that the ability of tumor cells to form clones and grow in soft agar was significantly reduced by crizotinib alone but decreased further following combined treatment. To address the question of cell death, we performed annexinV/7-AAD flow cytometry analysis. We found a clear induction of apoptosis upon crizotinib and chloroquine co-treatment when compared to untreated or single treatments, in both ALK-positive Karpas-299 and SU-DHL-1 cells (Supplemental Figure S7A–C and S7B–D, respectively, red quadrants). Of note, necrotic SU-DHL-1 cells were also detected (Supplemental Figure S7B, dark quadrant). Altogether, these results indicate that the reduced viability, soft agar growth capacity, and survival of ALK-positive Karpas-299 cells submitted to a combined ALK and autophagy inhibition strongly relies on the inactivation of a cytoprotective autophagy.

### Chloroquine treatment potentiates the growth inhibitory effect of crizotinib on Karpas-299 xenografted tumors

**Figure 5:**
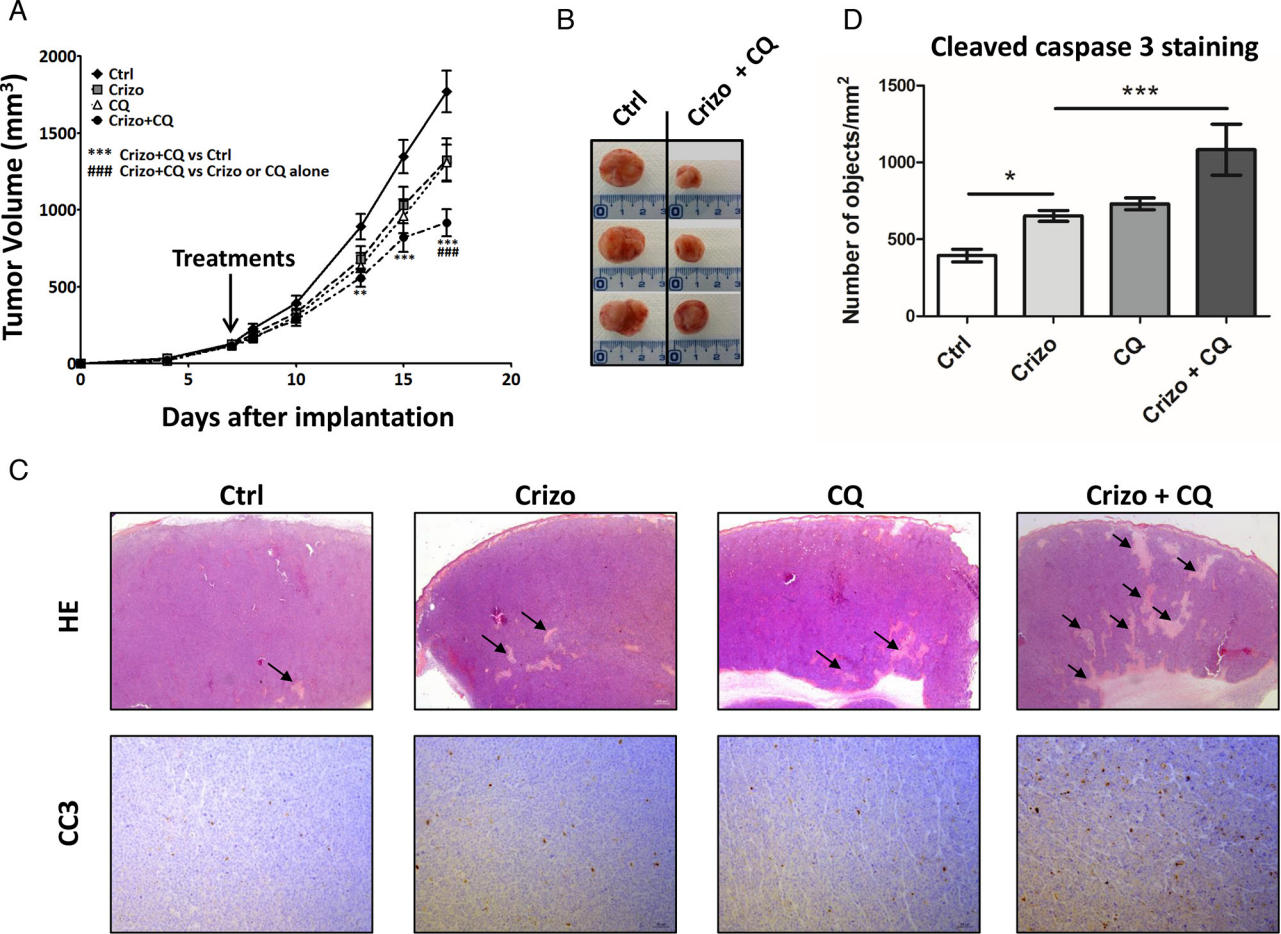
A combination of a low dose of crizotinib plus chloroquine inhibits Karpas-299 xenograft growth in NOD/SCID mice **A.** Karpas-299 subcutaneous tumors (*n* = 8 for each condition) were allowed to grow and, when measurable (around 100 mm^3^), mice were matched for tumor volumes and randomly assigned to receive crizotinib (Crizo, 10 mg/kg), chloroquine (CQ, 60 mg/kg) or a combination of both drugs (Crizo+CQ). Tumor volumes are reported as mean ± S.E.M. Statistical analysis was performed by two-way ANOVA using the Bonferonni correction; **p < 0.01; ***p < 0.001; ^###^*p* < 0.001. **B.** Representative tumors resected from mice submitted to either vehicle (Ctrl) or 10 mg/kg crizotinib and 60 mg/kg chloroquine combined treatment (Crizo+CQ). **C.** Micrographs of hematoxylin/eosin (HE) staining (original magnification x 12.5) and anti-cleaved caspase 3 (CC3) immunohistochemistry staining (original magnification x 100). Photographs shown are representative of similar observations in three different control (Ctrl), crizotinib (Crizo), chloroquine (CQ) and crizotinib + chloroquine co-treated tumors (Crizo+CQ), harvested from NOD-SCID mice developing Karpas-299 subcutaneous tumor xenografts. **D.** Active caspase 3 in tumor sections as indicated in (C). Quantifications were performed on scanned immune-stained slides (Pannoramic 250 Flash digital microscope) using a Pannoramic Viewer and HistoQuant software. Segmentation of the detected objects and the calculation of their number per mm^2^ were automatically performed. Statistical analyses were performed on individual raw data using one-way ANOVA followed by the Newman-Keuls multiple comparison test; *p < 0.05; ***p < 0.001. Values are expressed as mean ± S.E.M.

Having shown that autophagy inhibition enhances the anti-tumoral activity of crizotinib in ALCL cell lines *in vitro*, we next investigated the effect of the drug combination on the growth of Karpas-299 tumor grafts *in vivo*. As seen in Figure 5A, mice treated with a combination of crizotinib and chloroquine exhibited a significant decrease in tumor growth compared to untreated mice or mice treated with each drug alone. Overall these findings demonstrate that chloroquine treatment enhances the efficacy of crizotinib *in vivo*, and that the combined therapy, which was well-tolerated in mice, efficiently reduces ALCL tumor growth. We next examined the effects of these drugs on tumor cell necrosis/apoptosis using hematoxylin/eosin (HE) and anti-cleaved caspase 3 (CC3) staining in xenografted tumor tissues (Figure 5C and 5D). As shown in Figure 5C, HE staining reveals that tumor necrotic areas (arrows) were more extensive in tumors submitted to crizotinib and chloroquine co-treatment than in untreated (Ctrl) or individually-treated (Crizo or CQ) tumors, despite the fact that tumors submitted to a combined treatment were smaller than the untreated ones (Figure 5A and 5B). Similar findings were obtained for apoptosis using CC3 staining as an indicator of apoptosis (Figure 5C). Remarkably, a significant increase in CC3 staining was observed in the subcutaneous tumors harvested from animals that had been given the combined treatment compared to the individual treatments (Figure 5D). It should be noted that necrotic regions of the sections were excluded for the quantification of CC3. Altogether, these results suggest that the induction of necrosis/apoptosis could account for the anti-tumoral effects of the crizotinib and chloroquine co-treatment, which corroborates the *in vitro* findings shown in Supplemental Figure S7 and supports a cytoprotective role for autophagy upon crizotinib treatment (Figures 3 and 4). We conclude that crizotinib and chloroquine in combination is highly effective for impairing *in vivo* ALCL tumor growth.

## DISCUSSION

In this study, we demonstrate that crizotinib induces autophagy in ALK-positive ALCL cell lines, a result that has never been reported before in this particular subset of lymphoma. Since autophagy inhibition (either by pharmacological inhibition or by an siATG7-mediated approach) potentiates the anti-tumoral activity of ALK inactivation (either by crizotinib treatment or by an siALK-mediated approach), our results indicate that autophagy could act as a survival mechanism in therapeutically-challenged ALK-positive ALCL cells. ALK has previously been linked with autophagy, in glioblastoma and in crizotinib-resistant NSCLC cell lines [64, 65], however in both of these studies the ALK tyrosine kinases (the full length ALK receptor in glioblastoma, and the EML4-ALK fused oncoprotein in NSCLC) were not the direct target of the therapeutic treatment. In the glioblastoma cell lines autophagy was induced following cannabinoid therapy (acting on cannabinoid receptors), and in crizotinib-resistant NSCLC cells, high doses (1 to 8 μM) of crizotinib were used in cells harboring a loss of EML4-ALK. Therefore, another target point of crizotinib in those resistant NSCLC cell lines might be considered. We thus believe this ALCL study to be the very first one to reveal the induction of autophagy upon both pharmacological and molecular NPM-ALK inactivation. Indeed, our *in vitro* results demonstrate that an autophagy response is mounted and activated shortly after either crizotinib treatment (at a plasmatic concentration equivalent to that found in patients being treated for ALK tumors [66, 67]) or after siRNA-mediated specific ALK inhibition in ALCL cells. We observed five complementary results that support an autophagic response following ALK inhibition: 1) increased AVOs; 2) increased number of autophagosomes as identified by electron microscopy; 3) increased LC3-II immunohistochemistry staining and relocation to autophagosomal membranes; 4) increased autophagy flux (with LC3-II accumulation observed via western blotting following ALK inactivation and chloroquine co-treatment); 5) increased expression of autophagy genes. To decipher whether this crizotinib-induced autophagy affected cell death or cell survival functions, we performed several assays, testing cell viability, clonogenic survival, apoptosis and the ability of ALCL cells to form xenografted tumors *in vivo*. Together these assays demonstrated the cytoprotective action of autophagy following ALK inactivation in ALCL. Indeed, we found that: 1) upon combined ALK and autophagy pharmacological inhibition (using crizotinib and chloroquine or 3-methyladenine), these drugs had a synergistic (and not just an additive) effect on the reduction of cell viability; 2) the molecular inactivation of autophagy using siRNA directed against ATG7 did not induce, *per se*, a loss in cell viability, as was recently highlighted by D.A. Gewirtz [68]. It is noteworthy that a potentiating effect of autophagy and ALK co-inhibition was still observed following ATG7 downregulation; 3) ALK inactivation combined with autophagy inhibition drove cells towards apoptotic/necrotic cell death; 4) combined crizotinib and chloroquine treatment strongly reduced ALK-positive Karpas-299 clonogenic survival, unequivocally proving that autophagy harbors cytoprotective functions [68], and impaired xenograft tumor growth.

Altogether, these results strongly suggest that a combination of ALK and autophagy inhibition could be beneficial for the treatment of ALK-dependent ALCL, a therapeutic combination that has never been considered before. ALK-positive ALCL patients are currently treated with aggressive chemotherapy (cyclophosphamide, hydroxydoxorubicin, oncovin and prednisone (CHOP)). The use of crizotinib for ALK-positive ALCL is currently under debate. A good response to crizotinib has been reported in a few adult patients with recurring ALK-positive ALCL, as well as in one phase I clinical trials [17, 69, 70]. Furthermore, a recent clinical study using crizotinib as a monotherapy was performed on eleven adult ALCL patients who were resistant/refractory to cytotoxic therapy [18]. Their results, showing that crizotinib exerted a potent antitumor activity with durable responses and a benign safety profile, encourage the future use of crizotinib as a front line therapy. However, ALK mutations conferring resistance to crizotinib have been identified in relapsed patients. Thus, in light of our new data we propose that crizotinib-induced autophagy, through its cytoprotective function, could allow some cells to escape the targeted therapy and survive in a dormant state, as proposed by White and DiPaola [36] and as demonstrated in models of ovarian carcinoma and gastrointestinal stromal tumors (GIST) [29, 71–73]. This dormant state may be used by tumor cells to develop and acquire resistance to crizotinib, allowing subsequent tumor recurrence. Thus, in line with previous studies on imatinib-treated CML or GIST, showing that autophagy inhibition may represent a new strategy to enhance sensitivity to tyrosine kinase inhibitors, our current work supports the concept that crizotinib resistance and subsequent ALCL tumor relapse might be prevented or diminished by blocking autophagy. Nevertheless, before considering autophagy inhibition in ALCL patient therapeutic protocols, further clinical and fundamental investigations are needed to demonstrate that cytoprotective autophagy does indeed occur in human tumors upon crizotinib treatment and to provide a better understanding of how crizotinib mechanistically triggers autophagy induction. A possible mechanism could be inhibition of the mTOR pathway, which is known to be a negative regulator of autophagy [74], and which is reported to be activated downstream of NPM-ALK [75, 76]. Further studies on hydroxychloroquine (or new improved autophagy inhibitors) are also essential to determine the dose, frequency and treatment duration that should be used in patients to achieve autophagy inhibition [52, 77, 78]. Finally, besides ALK-dependent ALCL, this study should motivate further investigation into the effects of modulating autophagy in other ALK-related malignancies that harbor either different ALK fusions or overexpressed/activated full length ALK oncogenes.

## MATERIALS AND METHODS

### Cell lines and cell culture conditions

Karpas-299 and SU-DHL-1 ALK-positive ALCL cell lines bearing the t(2;5)(p23;q35) translocation were obtained from DSMZ (German Collection of Microorganisms and Cell Culture, Braunschweig, Germany). The FEPD ALK-negative cell line was a gift from Dr. K. Pulford (Oxford University, Oxford, UK). Cells were cultured in Iscove's Modified Dulbecco's Medium (IMDM) supplemented with 20% Foetal Calf Serum (FCS), 2 mM L-glutamine, 1 mM sodium pyruvate, and 100 U/ml penicillin/streptomycin (all from Invitrogen (Carlsbad, CA, USA) at 37°C with 5% CO_2_ and were maintained in exponential growth phase. This medium is hereafter referred to as “complete IMDM”.

### Chemicals

Crizotinib (Xalkori) was synthesized and purchased at @rtMolecule (Poitiers, France). Chloroquine (Aralen) (#C6628), 3-methyladenine (#M9281) and acridine orange (#318337) were purchased from Sigma-Aldrich (St. Louis, Missouri, USA). Stock solutions of crizotinib, chloroquine, acridine orange and 3-methyladenine were prepared in phosphate buffered saline (PBS).

### Small interfering RNA (siRNA) transfections

SiRNA transfections were performed by electroporation using Gene Pulser Xcell Electroporation Systems (Biorad) (Hercules, CA, USA). Briefly 5.10^6^ cells were electroporated at 950 μF to 250 V in 400 μl IMDM medium with 50 nM Beclin-1 siRNA, 100 nM ATG7 siRNA or 100 nM ALK siRNA from a 100 μmol/l stock solution or with the same quantity of a negative control siRNA (Eurogentec) (Seraing, Liège, Belgium). SiRNA sequences used were 5′-CAGUUUGGCACAAUCAAUATT-3′ for Beclin-1, 5′-GGAGUCACAGCUCUUCCUUTT-3′ for ATG7 and 5′-GGGCGAGCUACUAUAGAAATT–3′ for ALK. Following shock, cells were rapidly resuspended in 5 ml IMDM supplemented with 20% FCS. They were subsequently used for protein extraction, flow cytometry and viability/proliferation assays.

### Detection of acidic vesicular organelles (AVOs) with acridine orange

AVOs were quantified by flow cytometry. ALK-positive (Karpas-299 and SU-DHL-1) and negative (FEPD) cells (10^5^ cells), were treated or not for 24 h with crizotinib (500 nM and 400 nM, respectively), in the presence or absence of 10 mM 3-methyladenine (3MA) (added 4 h prior to harvesting the cells), or were transfected with scramble siRNA or Beclin-1 siRNA. They were then stained for 17 min with acridine orange (AO), at a final concentration of 1 μg/ml. Cells were washed twice in PBS, then resuspended in 0.3 ml PBS and analyzed on a FACSCalibur from Beckton Dickinson, (NJ, USA) using FlowJo software.

### Electron microscopy

Karpas-299 cells, treated or not for 24 h with 500 nM crizotinib, were collected, washed twice with PBS and fixed in 2% glutaraldehyde in 0.1 M Sorensen phosphate buffer (pH 7.4) for 4 h at 4°C. Cell pellets were then embedded in low melting point agarose to obtain solid blocks. These were washed overnight in 0.2 M phosphate buffer then post-fixed for 1 h at room temperature with 1% osmium tetroxide in 250 mM saccharose and 0.05 M phosphate buffer. Samples were then dehydrated in a series of graded ethanol solutions, followed by propylene oxide, and embedded in an Epon resin (Embed 812, Electron Microscopy Sciences (Hatfield, PA, USA)). Ultrathin sections (70 nm) were prepared (Ultracut Reichert Jung (Vienna, Austria)) and observed with a transmission Hitachi HT7700 electron microscope (TKY, Japan) at an accelerating voltage of 80 kV.

### Immunohistochemistry

Sections from formalin-fixed and paraffin-embedded xenografted tumors were stained with hematoxylin and eosin. Immunohistochemical analysis was performed using antibodies directed against LC3b (Nanotools (Teningen, Allemagne) #0231–100; mouse mAb; clone 5F10; 1/100) and cleaved caspase 3 (R&D Systems (Minneapolis, MN, USA) #AF835; polyclonal rabbit Ab; 1/500). Karpas-299 cells, treated or not with 500 nM crizotinib for 24 h, were included in low melting agarose and then formalin-fixed and paraffin-embedded. Sections were immunostained with antibodies directed against LC3b (Nanotools) and nuclei were counterstained with hematoxylin. Antibody binding was detected with the streptavidin-biotin-peroxidase complex method (Vector Laboratories (Burlingame, CA, USA)). Pictures were taken using either a Leica DM4000B microscope (Wetzlar, Germany) or a Pannoramic 250 device (3DHISTECH) (Budapest, Hungary).

### Autophagy RT-PCR array

Karpas-299 cells were treated or not for 24 h with 500 nM crizotinib, then washed once with PBS and harvested. Frozen cell pellets were sent to SABiosciences (Hilden, Germany), where both the RNA extraction and the Human Autophagy RT^2^ Profiler PCR array were performed to study the expression profile of 84 key genes involved in autophagy. Amplification data (fold changes in C_t_ values of all the genes) were analyzed by the ΔΔC_t_ method. Data were normalized to controls (PBS-treated cells), assigned as 1.

### qPCR

Briefly, 1 μg total RNA was reverse transcribed in 20 μl using the Superscript II reverse transcription kit (Invitrogen) and random hexamers (Roche), according to the manufacturer's protocol. Reverse Transcription (RT) reactions were diluted 10 fold prior to qPCR. Amplification was performed in a total volume of 10 μl containing 5 μl of a SYBR Premix Ex TaqTM (Tli RNaseH plus), Bulk master mix (Takara), 1 μl forward and reverse primers (final concentration of 300 nM each), and 2 μl diluted cDNA. The forward and reverse primers were, respectively: CCTCGCCAAGTCTCAGACGC/CCCCACCGTTGCAGTACTCC for ULK1, GAGCGCCT CTTCTCCAGCAG/CAGCCTTTGCCGGTTCAGCC for WIPI1, AAGCAGCGCCGCACCTTCGA/CGCTGACC ATGCTGTGTCCG for MAP1LC3B, GGGAAGCCTT TGGCCTTGCC/CCACTTGGGCATTCCTGGGC for PIK3C3, and CAACGACCACTTTGTCAAGCT/CTCTCT TCCTCTTGTGCTCTTGC for GAPDH. Q-PCR cycling conditions were performed according to the manufacturer's protocol, using the StepOnePlus real-time PCR system (Applied Biosystems). Results were analysed with the StepOne software.

### Western blotting

Cells were lyzed in radioimmunoprecipitation assay (RIPA) buffer (20 mM Tris HCl pH 7.4, 150 mM NaCl, 4 mM EDTA, 1% Triton X-100, and 0.2% SDS) supplemented with phosphatase inhibitors (1 mM Na_3_VO_4_, 1 mM NaF) and 1 mM phenylmethylsulfonylfluoride (PMSF), purchased from Sigma-Aldrich, and protease inhibitor cocktail (Roche Applied Science) (Penzberg, Upper Bavaria, Germany). Protein lysates were fractionated on SDS-PAGE (10 or 15%), and transferred to a nitrocellulose membrane (Whatman) (GE Healthcare, Little Chalfont, England). Western-blotting was performed using LC3-B (Sigma-Aldrich #L7543), ATG7 (Cell Signaling Technology #2631), Beclin-1 (Cell Signaling Technology #3738), ALK (D5F3 XP, Cell Signaling Technology #3633), ULK1 (Cell Signaling Technology #4773), GAPDH (Millipore MAB374) and β-actin (Santa Cruz #7210) antibodies. Proteins were visualized using the Chemiluminescent Peroxidase Substrate-3 Kit (Sigma-Aldrich) or the ECL™ Prime Western Blotting Detection Reagent (Amersham Biosciences) (Buckingshire, UK).

### Cell viability assay and multiple drug effect analysis

Karpas-299 and SU-DHL-1 cells were counted and seeded in 96-well plates (10,000 cells/well, in 100 μl IMDM/20% FBS). Cells were incubated at 37°C in the presence of either increasing concentrations of crizotinib (0 to 2000 nM) or were transfected by siRNA targeting ALK; and either chloroquine (0 to 120 μM), 3-methyladenine (0.625 to 10 mM) or siRNA targeting ATG7, alone or in combination. Combination experiments were carried out at constant ratios. After 48 h, cell viability was assessed using the CellTiter 96 AQueous One Solution cell proliferation assay (Promega) (Fitchburg, Wisconsin USA). Drug combination analyses were performed following the median-effect method using the CompuSyn software (ComboSyn, Inc., Paramus, NJ, USA) [59]. Briefly, drug interactions were determined by calculating the Combination Index (CI). In this method, synergy is defined by a CI values < 1, an additive effect by CI = 1, and antagonism is defined by CI > 1. The results are shown on the Fa-CI plot where Fa represents the fraction affected by the drug tested.

### Soft-agar colony formation assay

Karpas-299 cells were treated with 500 nM crizotinib and/or 30 μM chloroquine or were transfected with Atg7siRNA or scramble siRNA and allowed to recover for 8 h before treatment with 500 nM crizotinib. After 16 h, 20,000 Karpas-299 cells from each condition were resuspended in complete IMDM containing 0.33% agar onto the top of an agar underlay (complete IMDM containing 0.5% agar). Cells were fed twice a week with 400 μl complete IMDM containing the appropriate drug. After 7 days, viable cells were stained for 2 h with complete IMDM containing 3-(4,5-dimethylthiazol-2-yl)-2,5-diphenyltetrazolium bromide (MTT) (0.5 mg/ml). Four different fields were then scored from each plate and colony numbers were counted using Image J quantification software (U.S. National Institutes of Health, Bethesda, MD, USA). Experiments were carried out in triplicate.

### Apoptosis measurement

Analysis of apoptosis was done using annexin V (AnnexinV-PE) and 7-amino-actinomycin (7-AAD) (BD Bioscience #559763) staining according to standard protocols, followed by flow cytometry using a FACSCalibur cytometer from Beckton Dickinson. Results were analyzed using FlowJo software.

### Murine xenograft model

Mice were housed under specific pathogen-free conditions in an animal room at a constant temperature (20–22°C), with a 12 h/12 h light/dark cycle and free access to food and water. All animal procedures were performed following the principle guidelines of INSERM, and our protocol was approved by the Midi-Pyrénées Ethics Committee on Animal Experimentation. A total of 4.10^6^ Karpas-299 cells were injected subcutaneously into both flanks of 6-week old female non-obese diabetic-severe combined immunodeficient (NOD-SCID) mice (Janvier Labs) (Laval, France). Mouse body weight and tumor volumes were measured three times a week (once a day at the end of the experiment) with calipers, using the formula “length × width^2^ × π/6”. Mice (4 per group) were treated 5 times per week (monday through friday) once the tumor volume reached 100 mm^3^. Mice received crizotinib (10 mg/kg) or H_2_O orally, and chloroquine (60 mg/kg) or PBS by intraperitoneal injection. At the end of the experiment, mice were humanely sacrificed. Subcutaneous tumors and adjacent inguinal lymph nodes were harvested and sections were fixed in 10% neutral buffered formalin for immunohistochemical analysis.

### Active cleaved caspase 3-positive cell quantification

Immunohistochemical-stained slides were digitized using a Pannoramic 250 Flash digital microscope (P250 Flash, 3DHISTECH). Whole slides were scanned using brightfield scan mode with a 20X/NA0.80 Zeiss Plan-Apochromat dry objective (Zeiss) (Oberkochen, Germany), and images were acquired with a two megapixel 3CCD color camera (CIS Cam Ref #VCC-FC60FR19CL, CIS Americas Inc., Tokyo, Japan), achieving a 0.39 μm/pixel resolution. Pannoramic Viewer and HistoQuant software were used for viewing and analyzing the digital slides, respectively (RTM 1.15.3, 3DHISTECH). A minimum of 8 annotations per slide covering 50% of the entire tissue were analyzed using the same profile with the following characteristics: noise reduction (median filter strength = 0), object definition (HSV: 137 < Hue < 226, 26 < Saturation < 189, 17 < Value < 41), no filtering by size and no object separation. These settings allowed the automatic segmentation of the detected objects and the measurement of the number of detected objects per mm^2^.

### Statistical analyses

Results are presented as mean values ± standard deviations (SD) from at least 3 independent experiments unless otherwise indicated. Determination of statistical significance was performed using the Student's *t*-test for side by side comparison of two conditions. Welsch's correction was applied when variances were significantly different. For the experiments comparing more than two conditions, determination of statistical significance was performed using one-way ANOVA followed by a Newman-Keuls multiple comparison test. Xenografted tumor growths were expressed as the mean ± S.E.M. Statistical analyses were performed using the two-way analysis of variance (ANOVA) followed by the Bonferroni test using GraphPad Prism 5 software (GraphPad software) (La Jolla, CA, USA). For all tests, *p*-values less than 0.05 (*), 0.01 (**) or 0.001 (***) were considered statistically significant.

## SUPPLEMENTAL TABLES AND FIGURES


