# Supplementary material for: Targeting autophagy enhances the anti-tumoral action of crizotinib in ALK-positive anaplastic large cell lymphoma
Source: Oncotarget. 2015 Aug 17;6(30):30149–64. doi: 10.18632/oncotarget.4999 (PMC4745787; doi:10.18632/oncotarget.4999)
Supplement: Supplementary file 1 [file oncotarget-06-30149-s001.pdf]

SUPPLEMENTAL TABLES AND FIGURES

Supplementary Table S1: Increased LC3 immunostaining upon crizotinib treatment

	Control			Crizotinib		
	Weak (%)	Moderate (%)	Strong (%)	Weak (%)	Moderate (%)	Strong (%)
Exp 1	57.6	28.3	14.1	18.6	31.4	50.0
Exp 2	56.8	30.9	12.3	20.9	37.2	41.9
Exp 3	54.1	31.8	14.1	23.3	33.7	43.0

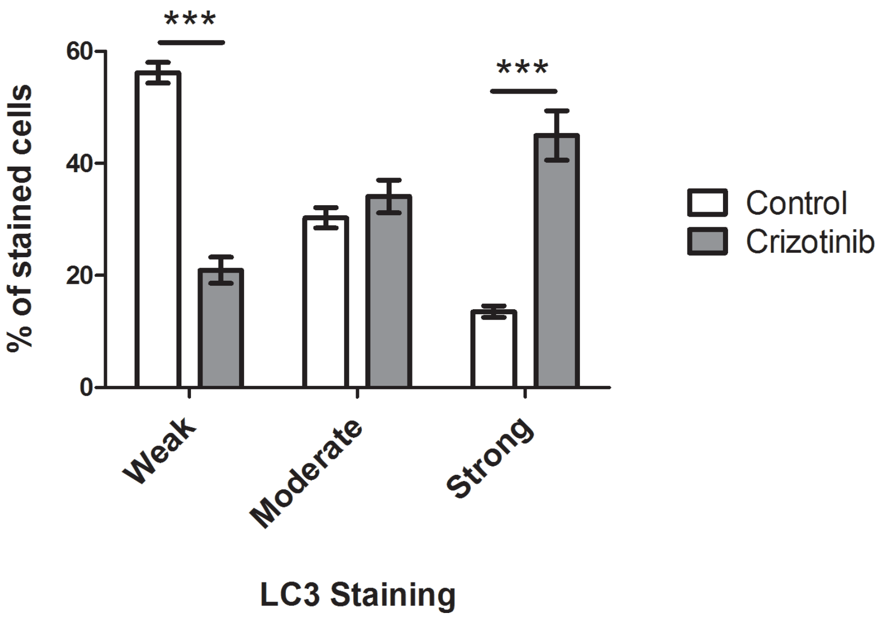

Immunohistochemical staining for LC3 was performed on crizotinib-treated (500 nM, 24 h) or untreated Karpas-299 cells (Control). Cells were counted and classified into three groups depending on the intensity of the staining. Within an observed cell, a weak signal corresponded to a diffuse staining, whereas a strong one corresponded to distinct LC3 puncta, indicative of autophagosome formation. The classification was performed on a total of approximately 100 cells, and by three different people. Mean values are shown in the graph below the table. Statistical analysis was performed by unpaired *t*-tests; $^{***}p \leq 0.001$.

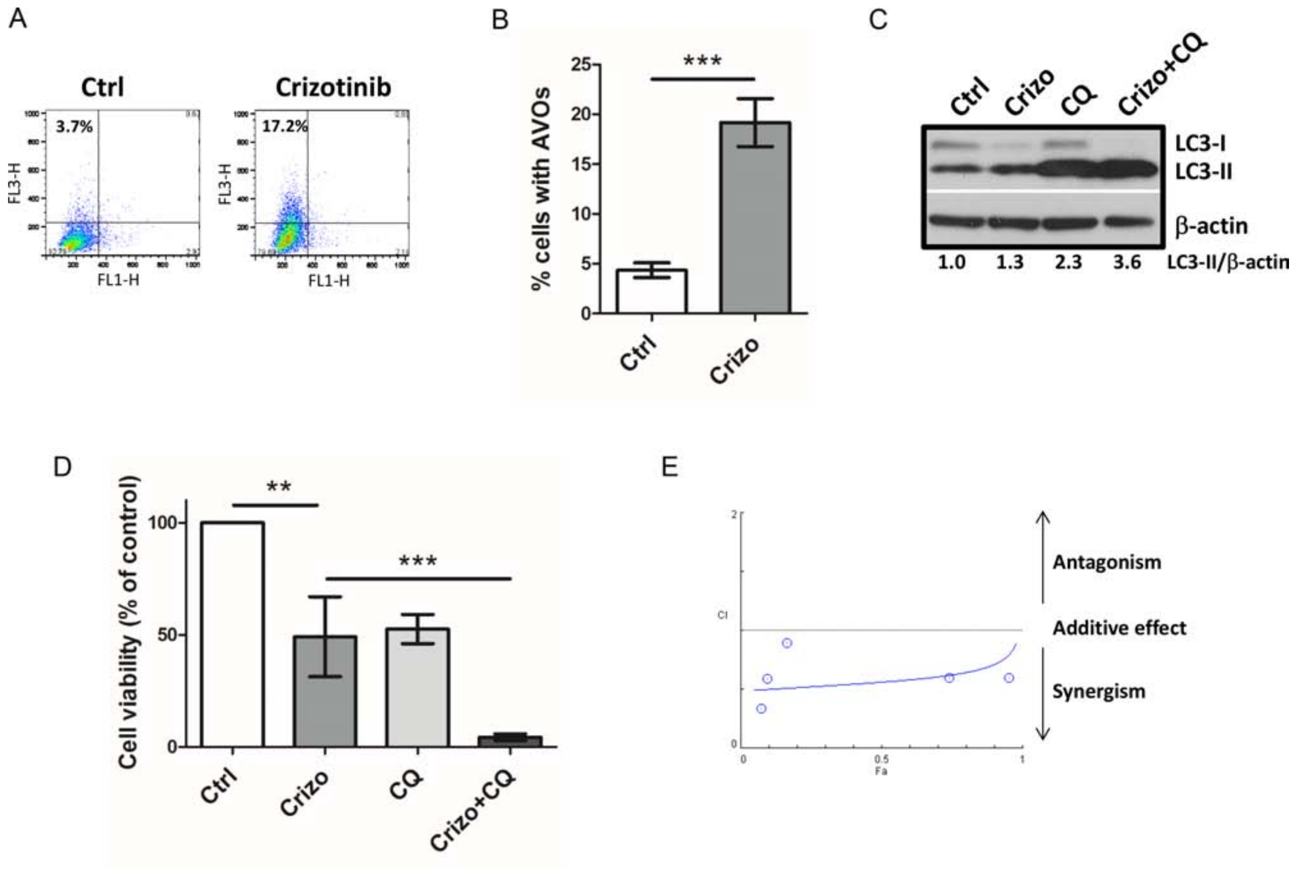

Supplementary Figure S1: Induction of cytoprotective autophagy in the SU-DHL-1 ALCL cell line by crizotinib. A. The formation of acidic vesicular organelles (AVOs) was determined upon crizotinib treatment (400 nM, 24 h) by the detection of red fluorescence in acridine orange-stained cells using flow cytometry analysis. The percentage of AVOs (left upper quadrants) is indicated. FL1-H indicates green color intensity (cytoplasm and nucleus), while FL3-H shows red color intensity (AVOs). Representative flow diagrams are shown. **B.** The development of AVOs (as indicated in (A)) was quantified by acridine orange FACS staining. The graph represents the mean values of three independent experiments ± SD. Statistical analysis was performed by unpaired *t*-tests; $***p \leq 0.001$. **C.** Autophagic flux was determined in SU-DHL-1 cells by treatment with crizotinib (400 nM, 24 h) (Crizo) in the presence or absence of chloroquine (30 µM, 24 h) (CQ). Total cell lysates were analyzed by western blotting using antibodies against LC3 and β-actin. Blots from one representative experiment are shown. The relative levels of LC3-II were normalized to β-actin. **D.** SU-DHL-1 cells were treated or not with crizotinib (400 nM) (Crizo) with or without chloroquine (30 µM) (CQ) for 48 h, then cell viability was determined by MTS assay. The graph represents the mean values of three independent experiments carried out in triplicate ± SD. Statistical analysis was performed by one-way ANOVA followed by the Newman–Keuls multiple comparison test; $**p \leq 0.01$; $***p \leq 0.001$. **E.** Cell viability data were analyzed and combination indexes (CI) were calculated using the CompuSyn software (ComboSyn, Inc., Paramus, NJ) for the determination of synergy. The results are shown on the Fa-CI plot where Fa represents the fraction affected by the drug tested. CI < 1 indicates synergism; CI = 1 indicates an additive effect, and CI > 1 indicates antagonism.

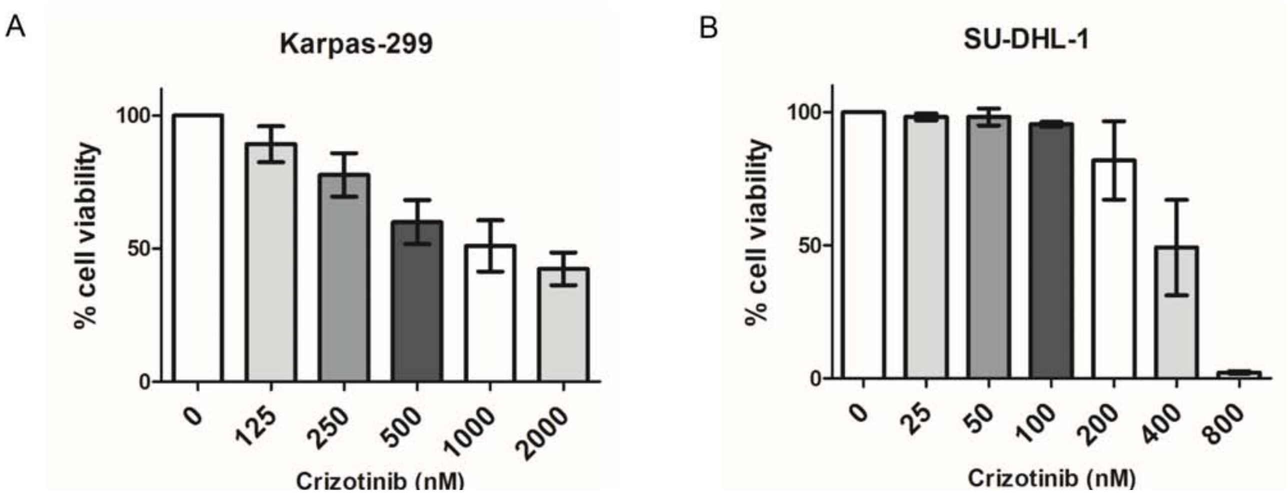

Supplementary Figure S2: Effect of crizotinib on Karpas-299 and SU-DHL-1 cell viability. A-B. Karpas-299 (A) and SU-DHL-1 (B) cells were treated for 48 h with the indicated concentrations of crizotinib (nM). Cell viability was measured by MTS assay. Experiments were done at least in triplicate and results were normalized to cells incubated without drug (100%). Data represent the mean values ± SD.

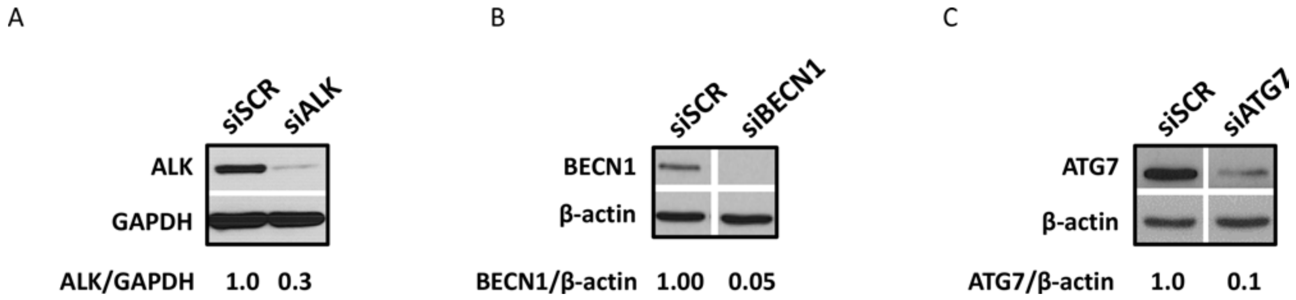

Supplementary Figure S3: Protein knockdown after siRNA transfection. A-C. Karpas-299 cells were transfected with scramble siRNA (SCR) or siRNA targeting *ALK* (A), *Beclin*-1 (BECN1) (B) or *ATG7* (C) for 72 h, 48 h and 72 h, respectively. The expression levels of ALK, Beclin-1 and ATG7 were then analyzed by western-blot. GAPDH or β-actin were used as loading controls.

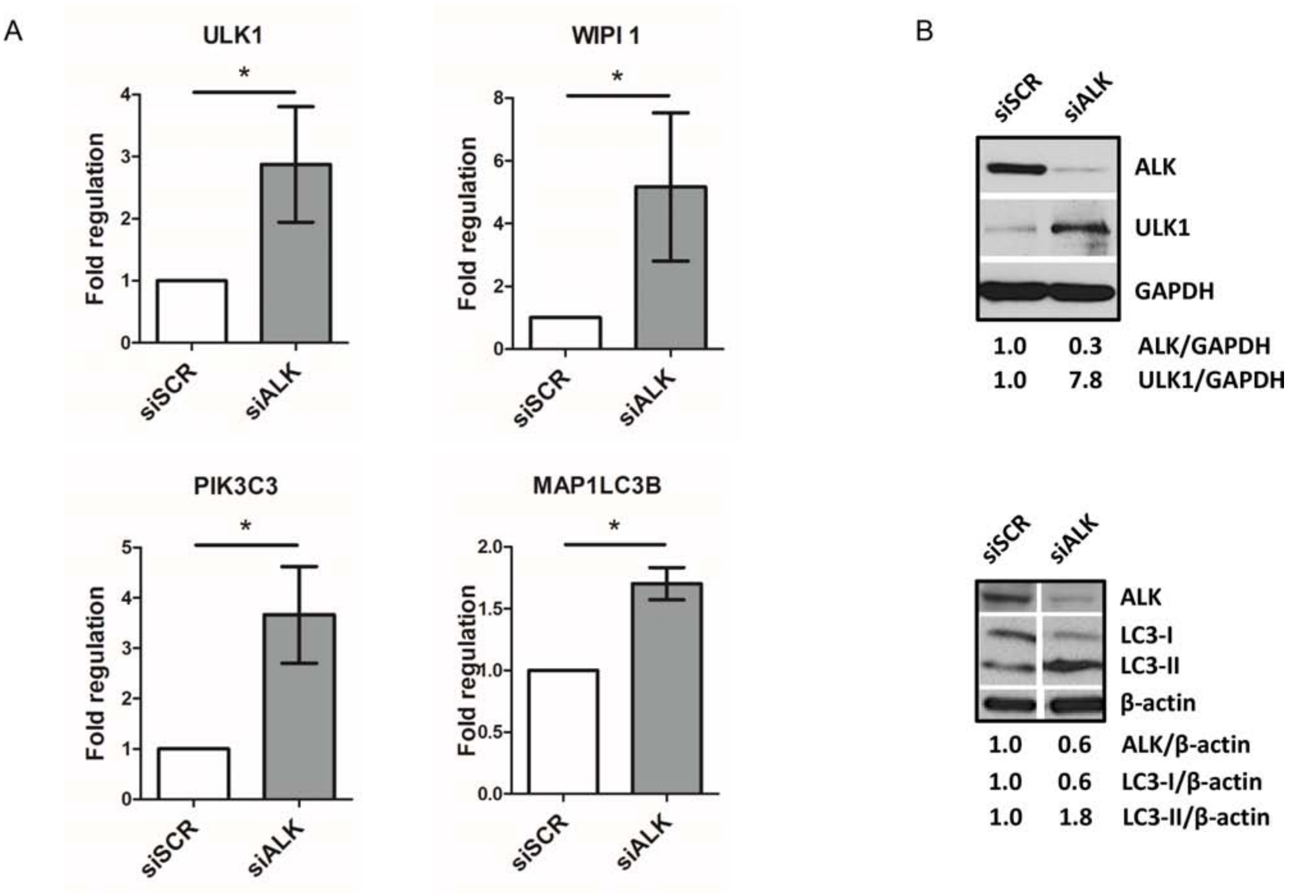

Supplementary Figure S4: Autophagy mRNA and protein upregulation upon ALK knockdown. A. ULK1, WIPI1, PI3KC3 and MAP1LC3B mRNA levels were determined by RTqPCR in Karpas-299 cells transfected with scramble siRNA (siSCR) and ALK-targeted siRNA (siALK). Values were normalized to the expression levels of the housekeeping gene GAPDH and are shown as fold regulation in mRNA expression compared to siSCR (set to 1). Statistical analysis was performed using unpaired *t*-tests; *$p < 0.05$. **B.** ULK1 and MAP1LC3B protein expression were analyzed by western blot with GAPDH or β-actin as loading controls.

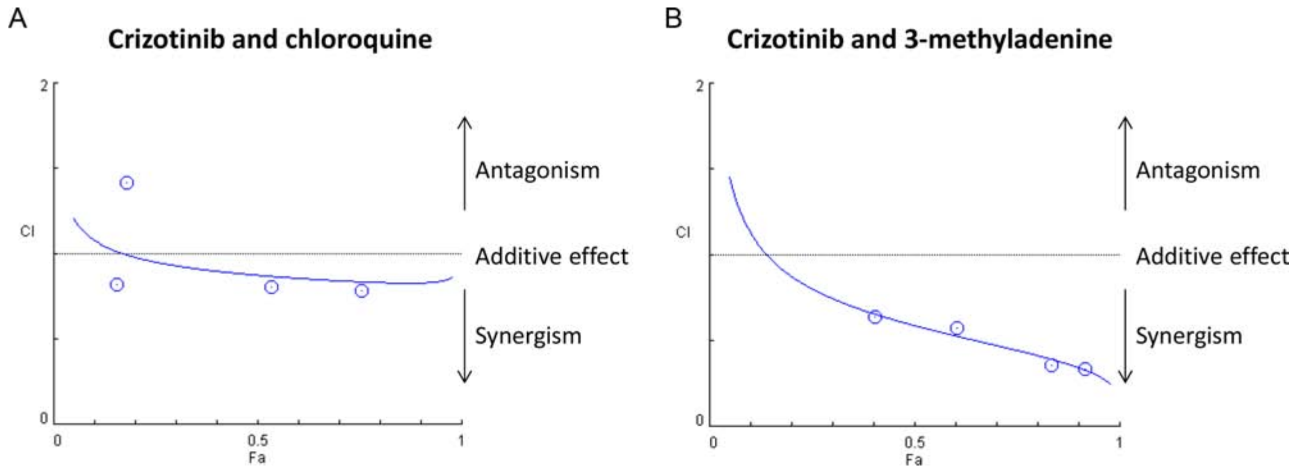

Supplementary Figure S5: ALK inhibition (with crizotinib) synergizes with autophagy inhibition (chloroquine or 3-methyladenine) to reduce Karpas-299 cell viability. Karpas-299 cells were treated with crizotinib and chloroquine **A.** or with crizotinib and 3-methyladenine **B.** simultaneously for 48 h (*n* = 3). The concentrations of crizotinib were (nM): 0, 125, 250, 500, 1000, 2000. The concentrations of chloroquine were (μM): 0, 7.5, 15, 30, 60, 120. The concentrations of 3-methyladenine were (mM): 0, 0.625, 1.25, 2.5, 5, 10. Cell viability data were analyzed and combination indexes (CI) were calculated using the CompuSyn software (ComboSyn, Inc., Paramus, NJ) for the determination of synergy. Results are shown on the Fa-CI plot where Fa represents the fraction affected by the drug tested. CI < 1 indicates synergism; CI = 1 indicates an additive effect, and CI > 1 indicates antagonism.

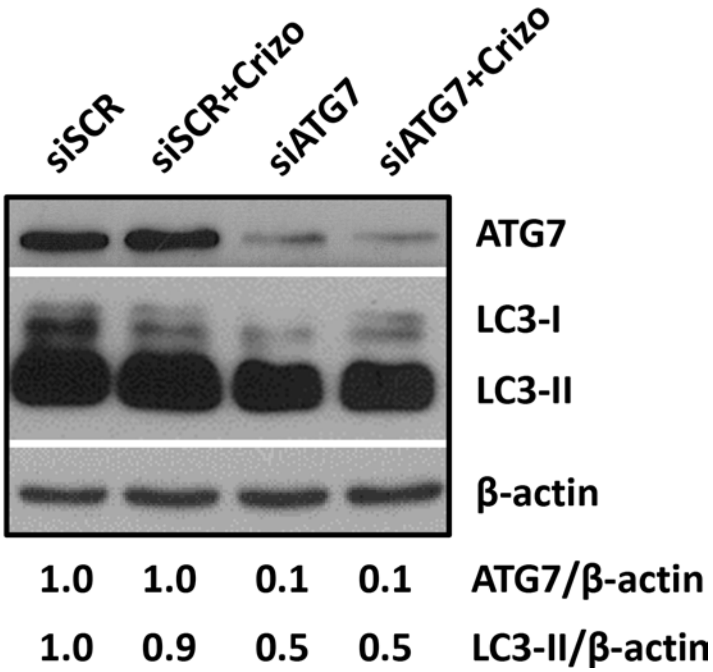

Supplementary Figure S6: Autophagic flux impairment using siRNA targeting ATG7. LC3 immunoblots were performed on Karpas-299 cells transfected with scramble siRNA (siSCR) or ATG7 targeting (siATG7) siRNA that were treated or not for the last 24 h with crizotinib (500 nM). β-actin was used as a loading control.

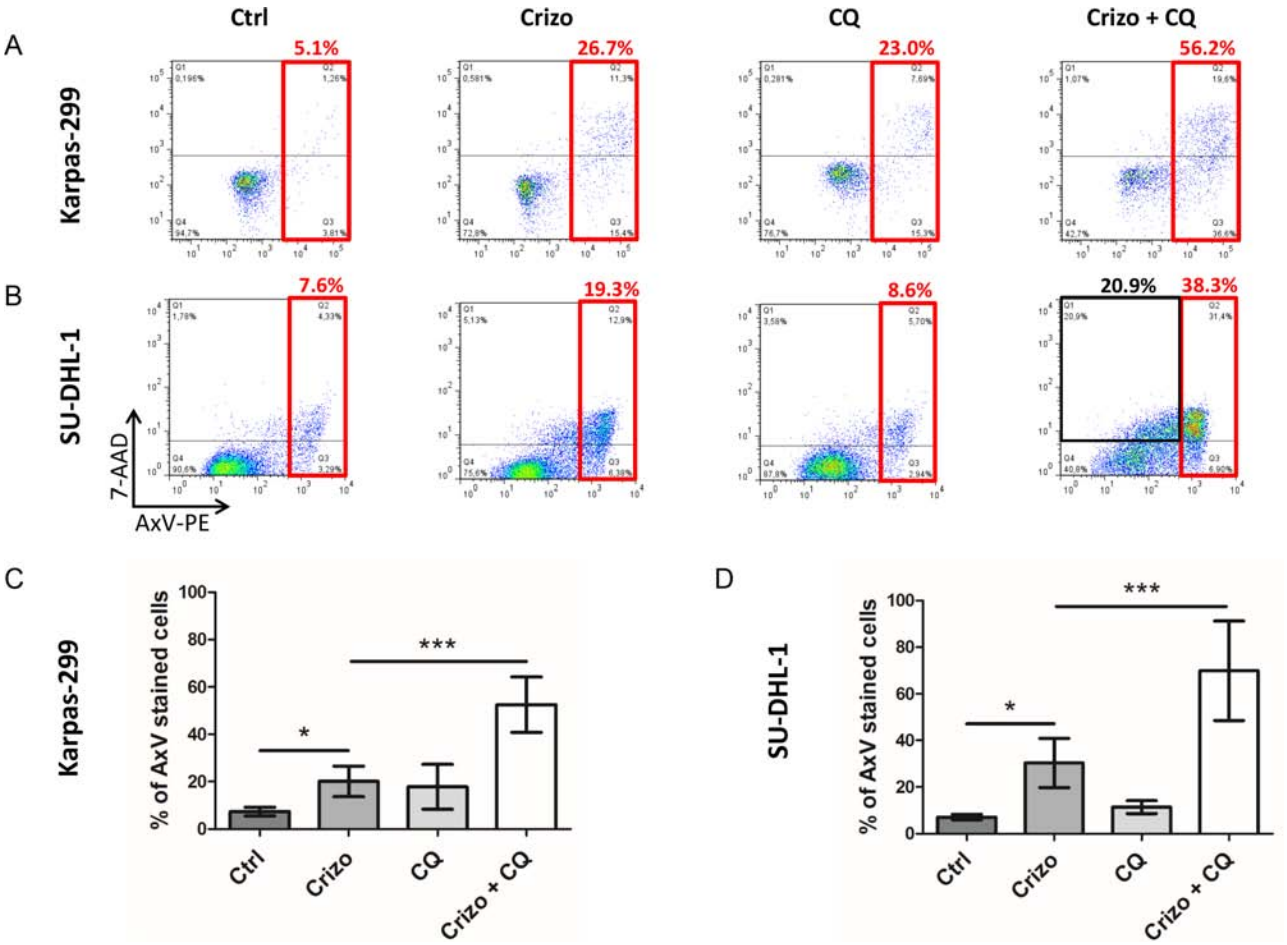

Supplementary Figure S7: Effect of crizotinib and chloroquine single and combined treatments on apoptosis. Karpas-299 **A, C.** and SU-DHL-1 **B, D.** cells were untreated (Ctrl) or submitted to crizotinib (Crizo) (500 nM for Karpas-299, 400 nM for SU-DHL-1) and/or chloroquine (CQ) (30 µM) single or combined (Crizo + CQ) treatment. Annexin V-PE / 7-AAD staining was performed 48 h after treatment. Representative flow diagrams are shown in A and B. The graphs in C and D represent the mean percentage of AnnexinV-stained cells from four independent experiments ± SD. Statistical analysis was performed by one-way ANOVA; *$p \leq 0.05$; ***$p \leq 0.001$.
